# Integrating Food Sensitive Planning and Urban Design into Urban Governance Actions

**DOI:** 10.1007/s12132-021-09417-9

**Published:** 2021-04-29

**Authors:** Gareth Haysom

**Affiliations:** African Centre for Cities, University of Cape Town, Cape Town, South Africa

**Keywords:** Urban food security, Food sensitive planning, Planning and urban design, Urban governance, Urban policy

## Abstract

Food access, stability and utilisation are key dimensions of food security at an urban scale. When the majority resided in rural areas, and lived predominantly agrarian lifestyles, it made sense for the state to govern food security through national agricultural ministries, focusing predominantly on the availability dimension of food security. With the transition to a majority urban world, coupled with the food security challenges currently experienced in urban areas, specifically in Africa, these historical policy and governance structures are increasingly inadequate in responding to essential food and nutrition needs. Problematically, urban areas, and specifically urban managers, cite unfunded mandates, and absent authority, as the reasons for not engaging food and nutrition governance responses. This paper argues that this is a false position. Drawing on recent data from household food security and poverty surveys, the paper calls for new and expanded planning and design approaches at the urban scale. The paper argues that spatial planning and urban design principles and actions provide an immediate and effective means through which to engage urban food system questions. Importantly these actions are essential to the transition from the current piecemeal project responses to urban food system inadequacies. Food sensitive planning and urban design is offered as a specific approach that could assist in programming food system–related challenges at the urban scale, responding to conceptual, analytical, organisational and design related dimensions of planning, and in so doing offering a longer term, systematic response to urban food insecurity.

## Introduction

South Africa is predominantly urban, with more than two-thirds of its population residing in urban areas. These urban areas are diverse, ranging from the conurbation that is the greater Johannesburg, to smaller towns and even sprawling peri urban settlements, some of which are legacies of South Africa’s Bantustan and apartheid planning systems. Additionally, in South Africa many consume inadequate, nutritionally poor diets. Food insecurity is a structural issue, one that requires an encompassing policy and strategic planning response. The current largely agrarian focus of food security policy misses both the urban demographic profile, but also the changing nature of the food system. All spheres of government carry an obligation to progressively realise the Right to Food. For many South Africans, this right is yet to be realised. Problematically, South Africa’s policy architecture effectively mandates national and provincial governments the role of enabling food access, and as such, ensuring the attainment of the Right to Food. This means that for urban areas, programming food security responses, and ensuring the realisation of the Right to Food and Nutrition is seen as a so-called unfunded mandate.

This paper argues that this is a flawed perspective. It argues that local governments have powerful tools at their disposal to effectively ensure the realisation of the Right to Food, and food security. These tools, specifically spatial planning, are not currently understood to have a food component. Food Sensitive Planning and Urban Design (FSPUD) offers a unique way in which to grapple with the intersections between the issues of inadequate food access, food insecurity, the (urban) food system, connections to wider systems, infrastructure and wider governance.

The paper starts with a broad discussion linking different aspects of planning, urbanisation and infrastructure. In order to highlight the urgency and importance of a specific urban focus, the paper then provides some detail on the state of food insecurity in South Africa and Cape Town (with reasons for the selection of Cape Town as a case study site). In order to reiterate the importance of the right to food and the obligations placed on local government, the paper briefly expands on how the right to food applies at the urban or local scale. The paper then concludes with a brief overview of food sensitive planning and urban design. The paper does not attempt to offer specific action steps, but rather presents the concept of FSPUD in broad terms, leaving it to planners and urban managers in specific contexts to operationalise such activities. While using the term FSPUD, it is appreciated that planning and urban design are not necessarily the same thing. In South Africa, and in many other African cities, “spatial planning happens at a variety of scales through plans and policies prepared by planners and policy drafters. Urban design happens at a smaller, precinct scale that typically, doesn’t include policy” (Faragher, 2021).

## Linking food system issues to place and space, and design

The urban food system is finally garnering greater attention, specifically in the context of the intersection between negative urban food system–related outcomes, such as food insecurity, hunger, escalations in non-communicable diseases and persistent wasting, and urban function and form (Battersby & Watson, 2019). Despite the current absence of systemic urban food related considerations, cities and the food system have always been connected (See Steel, 2008). Food and cities connect in multiple ways, and yet, most policy and governance responses view cities simply, as recipients of food produced in rural or even peri urban areas. The flows of food into cities shape how markets function, how household purchase, prepare and consume food, but also other considerations such as waste, the food economy, both formal and informal and a variety of food-related considerations.

This blind spot to urban food issues has emerged as a critical and increasingly urgent challenge in the face of the Coronavirus, SARS-CoV-2 (COVID-19) pandemic. Not only has the veneer of urban food security been exposed as being flawed (Battersby, 2020), with many falling into deep hunger in a very short space of time (Spaull et al., 2020), the impact of co-morbidities, many dietary related, has raised significant questions about the state of South Africa’s food system and the impact of non-communicable diseases on COVID-19 cases. Because of the specific trajectory of SARS-CoV-2, spreading from the first reported cases in Wuhan, China, to other large, well-connected urban centres in the world, and then from there to smaller towns and finally to rural areas, “a pattern known as hierarchical diffusion” (Mosely, 2020), the high urban prevalence and the scale of serious illness in South African cities requires far greater attention.

Given the pre-existing development challenges, and those now presented by SARS-CoV-2, new forms of urban development, management and governance are urgently needed. Aligning with a broader Southern urban perspective, the view offered by Pieterse et al. (2015) holds very true for South African urban areas at this time, that cities offer a small space where innovation and the re-thinking of urban governance can be developed. Speaking of infrastructure specifically, Pieterse et al (2015) go on to suggest that the infrastructure that is developed in African cities in the next 20 years will define the developmental trajectory of the continent well into the future. The same applies to South African cities and South African development trajectories.

This raises important questions about the urgent need for new forms of urban governance and the processes needed to facilitate access to items of a public good. A key public good is food (May, 2017). Food, the food system and food system outcomes, as well as food and nutrition security, are seen by most urban governance actors in South Africa as an unfunded mandate, something that will either be addressed by the market—the private sector—or another sphere of government, generally national government. However, food touches almost every aspect of urban governance, policy and economy. Wayne Roberts argued this point 20 years ago:

More than with any other of our biological needs, the choices we make around food affect the shape, style, pulse, smell, look, feel, health, economy, street life and infrastructure of the city.

(Roberts, 2001: 4).

The traditional silos of local government mean that efforts to govern food through a *ministry of* *food*, or through a sub-unit within another department will not deliver the integrated food system changes necessary to drive a truly urban food agenda. One of the few urban governance departments, or functions, that spans and intersects with all departments is planning. Planning, by its very nature is forward looking, as opposed to being reactive. This means that planning is ideally suited to respond to the urban food system challenge in a strategic and long-term manner.

That food needs to be an essential consideration for planners was recently affirmed by the Food and Agricultural Organisation, a body that generally drives the ruralised orientation of the food security discourse. The position argued applies directly to South African cities (South Africa is 66% urbanised), that.

with the majority of people already living in urban areas – not only in large metropolitan areas, but also in secondary cities and small towns – a greater focus on urban planning as a way of influencing food systems development will be critically important

(Stamoulis et al., 2018: v).

This perspective confirms both the primacy of South African cities in the wider food system, but also the need for cities to pay far greater attention to food and the food system.

This view of city-focused food systems governance, including planning as a key governance action, is emerging as a global trend. One of the most prominent governance documents connecting the food system–related challenges and cities is embedded in the New Urban Agenda (NUA) (UN-Habitat, 2017). Within the NUA food is seen as part of the city—one of the first times that such a perspective has been offered by a global governance institution.

More widely a diverse collection of urban food governance actions are emerging. These ideas and concepts sit within a growing set of urban food–related research and positions and are being rapidly mainstreamed. Some of the more prominent urban food governance perspectives include urban–rural linkages (UN-Habitat, 2017; Vorley & Lançon, 2016), the city region food system (Blay-Palmer et al., 2018), the supermarketisation processes (Reardon et al., 2003) and even the re-emergence of earlier work relating to the nutrition transition and urbanisation’s impact on this transition (Drewnowski & Popkin, 1997; Popkin & Slining, 2013). A number of these debates drove the embedding of food within the New Urban Agenda (Battersby & Watson, 2019). However, this does not denote a universal urbanisation of the food question. The absence of urban food issues within the Millennium Development Goals and now the Sustainable Development Goals, across all goals, but specifically the urban goal (SDG 11) and the hunger goal (SDG 2) (Battersby, 2017; Fukuda-Parr & Orr, 2014) confirms this.

Cape Town has demonstrated a progressive and forward thinking approach to food. Cape Town was the first South African city to develop an Urban Agriculture Policy (City of Cape Town, 2007), but also initiated a city-wide Food Systems Study completed in 2014 (Battersby et al., 2014). Recently, the city Resilience Strategy embedded food system actions and plans in this important strategy (City of Cape Town (CoCT), 2019). How this gets expanded and integrated into wider urban governance and management processes is yet to be seen.

Centrally planned and governed national food security and food system plans have their place but are ineffective in responding to the food needs of an urban majority. It is only through cities engaging in urban food issues that effective, and contextually relevant responses will emerge.

Planning offers the essential entry point to engage the food system, from the city scale, and for effective planning and governance processes to emerge.

Understanding the nature of food insecurity and how many urban residents engage the food system assists in understanding some of the connections between the food system and the urban system. This in turn offers insights into how and where planning and urban design activities can start to engage urban food questions. To elaborate on this, the case of Cape Town is used: Given the history of engagement in urban food, there is a sensitivity to food related issues and questions. Secondly, recent surveys provide data which can be drawn on to compliment some of the below arguments.

### Food (In)security and Food System Actions in South Africa and Cape Town

Food insecurity has been described as an “invisible crisis” in that food insecurity of urban populations has remained a marginal concern at all levels of government, despite clear evidence of rapid urbanisation taking place in the South, and in South Africa (Crush & Frayne, 2010).

While different measures are used to define food security in South Africa, and with due recognition of the contestation and diversity of measurement tools used (see Haysom & Tawodzera, 2018), this paper draws on three sources to highlight the state of food insecurity in South Africa. The first is the 2019 Statistics South Africa (StatsSA) report *Towards* *measuring food security in South Africa: An examination of hunger and food inadequacy*, informed by the regular General Household Survey (GHS), and despite contradictory conclusions, offers a sense of household hunger. The second is the *South Africa Demographic and Health Survey (SADHS) 2016* (NDoH, StatsSA, SAMRC & ICF, 2019) and the third is the South African National Health and Nutrition Examination Survey (SANHANES-1) from 2013, offering a far more in-depth analysis of the state of both food insecurity and nutritional outcomes of South Africa. At this time (2021), there is no other comparable national survey offering insights into food insecurity, stunting, wasting, obesity and other diet-related non-communicable disease outcomes.

The Statistics South Africa (StatsSA) report recognises South Africa’s urbanisation profile, arguing that “urbanisation is also another force that places more demand on food. … Two thirds of the South African population reside in urban areas” (Statistics South Africa, 2019: 21). But rather than considering the issues relating to food access and the links between infrastructure, spatial inequality and informality (issues associated with urbanisation with associated data within the GHS), the primary reason provided for the increased food security challenge in urban areas, is argued that “as people urbanise fewer are directly involved in agricultural production” (Statistics South Africa, 2019: 21).

Using the 2015 Food Poverty line (FPL) of ZAR 441,00 (US$ 2500), the StatsSA 2019 publication reports more than a quarter (25.2%) of the population living below a food poverty line (Statistics South Africa, 2019). However, other organisations use more current measures to determine possible food security challenges. For example, the Pietermaritzburg Economic Justice and Dignity (PMBEJD) organisation’s Household Affordability Index uses the 2018 FPL figure of ZAR 547,00 (Pietermaritzburg Economic Justice and Dignity Group (PMBEJD), 2019: 1).^1^

When considering food alone, research carried out in Pietermaritzburg indicates that the cost of a “basic nutrition food basket” for a family of four is calculated to cost significantly higher than the food poverty line would indicate, at an estimated ZAR 2 326,21 per month (Pietermaritzburg Economic Justice and Dignity Group (PMBEJD), 2019: 1).

The 2016 South Africa Demographic and Health Survey disaggregates specific health data across children and adults, also detailing gendered differences in adults. According to the NDoH, StatsSA, SAMRC and ICF (2019), just over a quarter of children under age 5 (27%) are stunted, 3% are wasted, 6% are underweight and 13% are overweight. Only 23% of children between the ages of 6–23 months are fed a minimum acceptable diet. The NDoH, StatsSA, SAMRC and ICF (2019) reported that the prevalence of measured hypertension (as at 2016) had nearly doubled since 1998, from 25 to 46% amongst women and from 23 to 44% amongst men. In the case of diabetes 13% of women and 8% of men age 15 and older were diabetic with very high proportions of women (64%) and men (66%) reported being pre-diabetic. The SADHS reported that 27% of women were overweight and 41% obese, while 20% of men were reported to be overweight and 11% are obese (NDoH, StatsSA, SAMRC & ICF, 2019).

The SANHANES-1 report, while more dated (2013), provides useful information on where hunger and food system–related issues are experienced. According to SANHANES-1, 37% of respondents experiencing hunger were in the rural formal sector, but 32% were in urban informal areas (Shisana et al., 2013). The highest prevalence of the population at risk of hunger was in urban informal areas (36%). The residents of these areas are mainly of low economic status who are largely unemployed, or if not, earn low, irregular incomes and struggle to provide the basic necessities. Importantly, given the far higher South Africa population living in urban areas, the use of proportions in the SANHANES data masks the actual net numbers of hungry in urban areas.

Food insecurity is not simply vulnerability to hunger, but also consumption of nutritionally deficient diets. The SANHANES Survey indicated that about 27% of boys and 26% of girls from 0 to 3 years of age are chronically malnourished (Shisana et al., 2013). While malnutrition persists, overweight, obesity and diet-related non-communicable diseases, such as diabetes, are on the increase, as highlighted in the DHS. Other nutritional issues were also found. Anaemia amongst adult women in South Africa was found to be 31% (NDoH, StatsSA, SAMRC & ICF, 2019).

These findings are consistent with the changes in diet known as the nutrition transition. These dietary shifts are in part the result of urbanisation and the time scarcity of urban life, and the desirability of a “modern” diet, but they must also be understood as the outcome of the changing food system and the unaffordability of healthy foods, linked to urban system functions (Battersby et al., 2014).

Poor and inadequate food and nutrition security cannot be viewed as simply an issue of food alone. Far wider systemic questions require attention. One of the clearest demonstrations of these connections comes from the Pietermaritzburg Economic Justice and Dignity organisation. According to PMBEJD data, when associated household costs are considered, including transport to and from work, insurances, school fees, related services costs, such as water, sanitation and energy costs, hygiene products, etc. the required monthly household income for a family of four who could afford a basic nutritionally adequate diet is R7 624,13 (US$ 435) (Pietermaritzburg Economic Justice and Dignity Group (PMBEJD), 2019: 11).

Clearly poverty as a proxy for food security offers some insight into the possible instances of food insecurity, but to fully understand food security, and plan policy and programming responses that ensure change, a far more encompassing approach is required. When these factors are considered in the context of how infrastructure and services impact food security, effectively casting that impact in concrete for the next 30–100 years, far greater consideration, and urgency in policy and planning, is required. This attention also needs to be linked to place—where the subsequent multi-dimensional drivers of food insecurity and place intersect in what is referred to as the food environment. The limited “poverty equals possible food security” lens has problematic implications as the poverty focus obscures any consideration of the wider systemic factors that impact food security. Building on the PMBEJD and recent increases of food insecurity that emerged as a result of the Coronavirus SARS-CoV-2 lockdown, it is argued that the Statistics South Africa (2019) figures grossly under-estimate food insecurity. The StatsSA figures may capture a measure of hunger, but hunger and food insecurity are very different. The SADHS and SANHANES figures offer stark insights into the nutritional deficits and the resultant dietary-related consequences of these deficits. These are issues that play out differently at different scales. Disregarding one of these scales, particularly the urban scale, leaves significant gaps in the policy and planning responses.

Findings from surveys using the US Aid Food and Nutrition Technical Assistance (FANTA) survey tools, specifically the Household Food Insecurity Access Prevalence Scale (HFIAP), are used here to qualify claims of high levels of food insecurity. In 2008, the African Food Security Urban Network (AFSUN) investigated the state of food insecurity in predominantly poor areas of eleven African cities and found that 80% of the poor communities surveyed were food insecure (Frayne et al., 2010). In Cape Town, 80% of the poor households surveyed were either moderately or severely food insecure, a figure that rose to as high as 89% in Khayelitsha (Battersby, 2011: 13).

A more recent 2013 Hungry Cities Partnership survey, using the same FANTA tools, sought to engage the wider city. In Cape Town, 49% of all respondents were either severely or moderately food insecure (Crush et al., 2018). However, when read across income quintiles, the figure presents a challenging trend, one that reflects a far more generalised food system failure, demonstrating how vulnerable many communities are to food insecurity, not just those at or below the poverty line. Table 1 demonstrates the state of food (in)security in Cape Town, detailing not just the food security scores (HFIAP) across income quintiles, but also variations in dietary diversity (HDDS) where a figure of less than 6 serves as a proxy for under-nutrition, and months where household food managers feel that they are unable to adequately provide for their households (MAHFP).

**Table 1:** Broad suite of household food security and dietary outcomes across income terciles (*n* = 2504) (source: Crush et al., 2018—from 2013 data)

		Income quintiles				
		1	2	3	4	5
HFIAP	Food secure	8.1%	21.7%	32.7%	66.2%	91.9%
	Mildly food insecure	5.1%	6.7%	8.9%	6.9%	1.7%
	Moderately food insecure	16.9%	19.5%	18.9%	9.2%	1.7%
	Severely food insecure	69.8%	52.0%	39.5%	17.7%	4.7%
HDDS (0–12)	Mean scores	5.1	6.2	6.7	7.5	8.0
MAHFP (1–12)	Mean scores	7.1	8.8	9.7	11.0	11.9

The Household Food Insecurity Access Prevalence Scale (HFIAP) and the Household Dietary Diversity Score (HDDS) as well as the Months of Adequate Household Food Provision (MAHFP) are part of a suite of food security measures developed by US Aid Food and Nutrition Technical Assistance (FANTA) measures (Swindale & Bilinsky, 2006; Coates et al., 2007; Coates, 2013)

Table 1 demonstrates what could be referred to as a continuum of food insecurity across income quintiles, where extremes may dissipate from quintile 1 to perhaps even 4, but food insecurity and vulnerability exist, across most incomes, none the lowest quintiles. Table 1 also offers insight into the reasons why, when the SARS-CoV-2-related lockdowns were activated, so many fell from vulnerability into hunger in a short period of time.

**Fig. 1:**
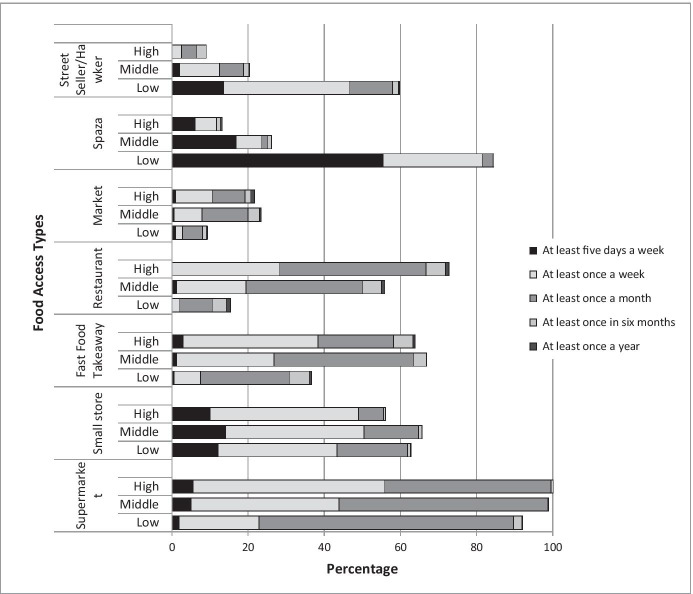
Household food access strategies by income tercile—Cape Town (Source: Crush et al., 2018) (n = 2504)

Other questions need to be asked about how the food insecure access food and what could be described as their ‘purchasing profiles’. Figure 1 draws from the 2013 Cape Town HCP household survey. This demonstrates the manner in which the food system is used by different income groups.

While most households accessed some food through the formal supermarket system, for the poor, this was mostly on a monthly basis. From qualitative interviews, this practice was explained as being due to supermarkets being used to access key staples, generally non-perishable foods, where buying in bulk offers benefits. These are the food types that can be easily stored for extended periods of time. For most of the daily and more frequent purchases, the informal sector is central to the food access strategies of the poor, where over half the lowest income tercile respondents mentioned frequenting spaza shops at least five times a week, and a large proportion of these same respondents reported frequenting street vendors on a weekly basis (Crush et al., 2018). “For most things, they come here every day, so our items are small, for use on the day” (Respondent 4.23.7, 2019).

Clearly while the supermarket is frequented and used by most Capetonians, other food access points are also used, and used very differently, often in response to home and neighbourhood infrastructure provision. Food choices correlate directly to the lived reality of households. While income frequencies (monthly, weekly or daily) would influence this, other factors such as access to storage, energy access and its reliability, and refrigeration all drive food choice and choices about the point of food access. Clearly, many factors beyond those of just income poverty, contribute to food insecurity and impact food access. These factors are linked to infrastructure, spatial planning and urban design.

**Fig. 2:**
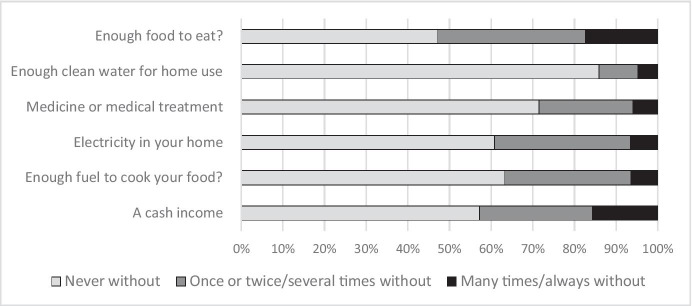
Lived Poverty Index results from Cape Town (survey from 2013). Responses to question “How many times did you go without …?” (source: from Crush et al., 2018)

Figure 2 presents the results drawn from the multi-dimensional poverty tool, the Lived Poverty Index (LPI), used in the same Cape Town survey (Crush et al., 2018). The LPI includes all income categories, and as a result, is mediated by the very regular access to services of the wealthier income groups. The LPI highlights the fact that access to income remains a challenge. This is however not the only limitation faced by these households. Over and above income poverty, the LPI provides insights into other drivers of poverty, drivers that have a direct impact on food security.

Access to clean water is essential in providing safe and nutritious food (the utilisation dimension of food security). Over 15% of respondents state that they “never went without” water. It is worth highlighting though that these figures reflect a scenario that pre-dates the 2017/18 drought in Cape Town. Current research in poor settlements in Cape Town indicates that water is accessible but regular access is constrained as a result of the “choke” system that was introduced as a water-use control measure during the drought.^2^

Households also reported high levels of inadequate access to food (Fig. 2), markedly higher than the official Statistics South Africa (2019) figures. While over 42% of respondents reported inadequate incomes, this figure needs to be read in the context that despite income and food deficiencies, households also experienced deficiencies in access to electricity in the home (with 32,6% reporting some disruptions and a further 6.6% reporting frequent lack of access). But food is not cooked using electricity alone. In the Cape Town 2013 survey, just under 37% of respondents reported some form of limited access to energy to cook their food (Crush et al., 2018). These infrastructure aspects, all reported at the household scale, impact household food related outcomes directly.

Constrained access to energy, both electricity and the different forms of cooking fuel, mean that households make strategic decisions on the types of foods prepared, often resorting to more processed and faster cooking food types. A further essential consideration relating to electricity access is its role in food preservation. Without electricity refrigeration is generally absent. This means that households strategically orientate their food purchases around such limitations. As a result, foods purchased often include processed foods that have a longer life (such as processed staples).

These strategies show three clear consequences; first as a result of the processed nature of the food, there is often an increase in food costs. Second, there is a potential reduction in nutrient value of that food. Third, ultra-processed foods also present health risks (Monteiro et al., 2010). Without access to refrigeration, the imagined benefits of the modern supermarket—be these bulk discounts, food safety and variety—cannot materialise.

Alone the electricity related infrastructure deficit has a profound impact on the food system of the households. When considering the wider food system, if traders and neighbourhood retailers do not have access to electricity, water, even toilet facilities, this impacts their operating, stocking and daily work practices and supply cycles. In the context of SARS-CoV-2, this is even more challenging. Infrastructure deficits impact the types of food stocked. Infrastructure impacts many factors including costs, hygiene and supply chain processes. A question seldom asked in the debates around the nutrition transition and rise of NCDs in South Africa is what role energy, water and other infrastructure access plays in this transition? Clearly, food insecurity is directly linked to place and factors other than just food production or poverty. This implicates different levels of the state to act and ensure access to food and nutrition.

### The Right to Food and Obligations of Different Spheres of Government

Despite being enshrined in the South African Constitution as a Right, as others have argued (De Visser, 2019), food is also a public good (see May, 2017). Such a description invokes notions of a duty of care and state centred obligations that require action from all state actors, coupled with processes within policy and governance that enable the fulfilment of this right and access to the public good.

In a time when the rural demographic environment dominated, there were clear and understood links between food security and agriculture. As society has urbanised, policies and governance mandates have not kept up with these changing demographics. South Africa is a particular case in point where the national Department of Agriculture, Land Reform and Rural Development (DALRRD) still retains the overall mandate for food security. Policy located within a department, whose primary mandate is food production, results in a particular view of food security needs, but also a predominant perception of where the food insecure may reside, the rural areas of the country. This maintains the centralised, national government governance mandate. Problematically, this perspective of the location and governance of food insecurity drives fiscal allocations, or budgeting, that facilitates action and governance authority.

Rights to Food and Nutrition are enshrined within the South African Constitution, in Sects. 27.1.b and 28.1.c. The South African constitution obligates all state entities to ensure the progressive realisation of the right to food (Republic of South Africa (RSA), 1996). This obligation does not rest solely on national and provincial governments; it also applies to and binds local government. This food security, and by extension food system, obligation is further reinforced by the food system–related obligations placed on urban areas, or local government, through schedules 4 and 5 of the same constitution (see De Visser, 2019; Republic of South Africa (RSA), 1996). However, despite this broader and encompassing obligation, the absence of both a policy mandate (as opposed to a legal mandate derived from the Constitution), and a fiscal mandate, through state provided funding to programme food security responses, means that food security remains a so-called “unfunded mandate” in the eyes of most local government actors (see Battersby et al., 2014).

Importantly, however, even without specific food security programmatic funding, the South African policy landscape offers city managers, politicians and bureaucrats opportunities to engage urban food questions far more deliberately than they do at present. As De Visser (2019: 25) suggests:

the Constitution allocates many functions to local government that offer points of leverage for municipalities to make meaningful contributions to the realisation of the right of access to food.

Central to De Visser’s argument is the role of planning in creating an environment in which local government can enact proactive food system–related responses pointing out that:

‘Municipal planning’ is one of the most critical local government powers. It is the power of municipalities to plan and manage the use of land, which is commonly referred to as ‘town planning’.

(De Visser, 2019: 14)

Drawing on De Visser’s (2019) argument and links to specific jurisprudence, specifically those relating to judgements made in the context of the realisation of specific entitlements enshrined within the South African Bill or Rights, a key tool that cities hold is through its planning regulations. This is important for two reasons: the first is that planning is a direct local government competence, secondly given the integrated vision of the current planning processes (Spatial Planning and Land Use Management Act (SPLUMA), 2013). A number of overriding planning principles could guide how planning and urban food system governance could be integrated. Considerations include:

- That residents have to access an adequate, healthy and appropriate diet
- That settlements are socially inclusive
- That settlements are equitable
- That settlements are more resource efficient
- That settlements are sustainable
- That planning caters for cultural difference

(Park Ross, 2019: 5).

South African cities have strategic tools and mechanisms which include amongst other things, the Integrated Development Plans and Spatial Development Frameworks, both of which need to engage with the urban food system in significantly more detail. A scan of many of these notes that city-scale strategic engagement in the food system is largely absent, bar some mention of urban agriculture projects or the “formalisation” of hawkers and traders. In South Africa, planning is implicated in the emergence of unsustainable and unhealthy urban food system patterns, while at the same time, planning has the tools to facilitate more inclusive and nurturing food system responses (Battersby, 2017). However, it remains largely ignorant of its deep impacts on the food system (Battersby, 2017). The absence of a definitive, proactive, integrated and pro-poor strategy focusing explicitly on the informal sector in Cape Town is a key example of the absence of deliberated food system planning (Skinner & Haysom, 2016). It is through planning that opportunities for a more inclusive food system lie, where the integration of direct food system interventions and the embedding of food considerations into planning decisions are most evident. These needs are essential because “improved access to and utilisation of food is essential to current and future generations. With cities at the centre of our civilisation, it will become increasingly critical for food to be centrally reflected in the planning of urban areas” (Stamoulis et al., 2018: vi).

### Food Systems Planning and Urban Design

Despite the lack of consideration for food within the planning field, urban planners unknowingly already play a central role in almost every component of the urban food system, as well as the wider regional food system, directly influencing the flows and a variety of other systemic activities that intersect with the food system. The impact of such planning decisions are often negative given that “decisions that impact the food system are made without consideration of the food system consequences” (Park Ross, 2019: 6).

Three food system trends dominate current scholarly discussions; an increasing role played by supermarkets, nutrition changes taking place, particularly those aligned to urbanisation, and the so-called big food transition. These are all linked and intersect with one another in ways that we do not fully understand. These interrelated processes are affecting the nature of food systems and food insecurity in South Africa (Haysom, 2015). Supermarkets in particular play both a dominant role in the supply chain, but are also changing the food retail landscape of cities (Peyton et al., 2015; Reardon et al., 2003; Tschirley et al., 2013). In addition, supermarkets are important sites of property transactions and development, which can often undermine other food retail livelihoods in urban areas (Teppo & Houssay-Holzschuch, 2013). Changes in food consumption are associated with how urban space is used in terms of travel time constraints leading to dietary changes, driving a nutrition transition (Popkin, 1998). The “big food transition” is linked to the preceding transitions, and to wider agro-food system changes (Igumbor et al., 2012; Monteiro & Cannon, 2012), suggested by Hawkes (2006: 1) to be “the convergence towards poor quality obesogenic diets”. The forces of urbanisation collide with rapid changes taking place within the food system, exacerbated in the manner in which urban planners respond to the urbanisation question.

Arguing that food and planning are co-dependant implies that planning and food system processes could align to generate positive societal outcomes. While this may be true, exactly how this is programmed is yet to be fully understood. Planning academics have started arguing for greater food system engagement within the planning field (Morgan, 2013; Pothukuchi & Kaufman, 1999; Sonnino, 2009) and while conceptually relevant, practically, very little change is evident in both the urban governance and urban planning and design fields.

Different case studies reflect different forms of planning for food system activities. Importantly, it is not just Northern cities that have been engaging in food-aligned planning actions. A scan of literature and concepts demonstrates real innovations in Southern cities (Park Ross, 2019), from planning aligned to urban growth (Zhong et al., 2018), to overall city-scale food system engagement across multiple development challenges (Rocha & Lessa, 2009), to context specific processes to address an area of particular development importance (Dobson & Skinner, 2009). Nascent processes are evident but how can planners effectively engage the urban food system?

Water Sensitive Urban Design (WSUD) as a concept was first devised in 1990s in Australia and arose as a response to the challenge of how to design for resilience as a result of the converging impacts of population growth and climate change, specifically in relation to the need for sustainable urban water resource management and the protection of water habitats and environments (Wong & Brown, 2009). This was in the context of severe water quality, quantity and drainage challenges in Western Australia (Gluckman, 2017). The conceptual entry point was the assertion that the overall water system, and its complexity, had been disregarded in favour of a disproportionate focus on perfecting efficiency of only one dimension of the system, around water supply (Wong & Brown, 2009). WSUD positions water as the driver for the planning of ecologically sustainable cities and aims to optimise the synergy between the urban built environment and the urban water cycle. The same argument is now being made in respect of food, specifically urban food.

One of the core texts setting out the concept of Food Sensitive Planning and Urban Design (FSPUD) engages the current urban challenge as one where cities are facing unprecedented change, across multiple governance domains. Donovan et al. (2011) articulated this as the converging and mutually compounding threats of climate change, vulnerability to peak oil, loss of land and resource scarcity, but sought to respond to these through the concept and principles embedded within the aligned practices of urban planning and governance.

Their work is novel and is applicable across cities of the North and South setting out new ways of “tackling issues, providing a suite of ideas and innovations that cities should now embrace … It tackles a topic that has little precedent as an agenda for the planning of cities” (Donovan et al., 2011: 2). From this the claim is made that “[t]his approach will not only improve the liveability of our cities, but will also deliver a more sustainable food system” (Donovan et al., 2011: 2). The principles and overarching concepts within the Food Sensitive Planning and Urban Design approach offer a useful starting point to engage what this may entail in the South African context. Clearly, given the South African (and African) context issues of redress and greater equity need to be included here, something perhaps missed given the developed world origins of the FSPUD concept. Despite these limitations, food sensitive planning and urban design can be reconciled with the aspirations of planning and urban design:

- Making sure we can enjoy attractive, liveable surroundings.
- Facilitating a strong and competitive economy.
- Facilitating major reductions in the environmental footprint of our settlements.
- Providing opportunities for stronger community interactions.
- Ensuring better shared spaces.
- Supporting fair access to the appropriate goods and services people need.
- Supportive environments for active living making sure these qualities can be provided indefinitely and are resilient to challenges such as peak oil and climate change.

(Donovan et al., 2011:11).

Central to the FSPUD concept is that it is embedded in planning and urban design. This may seem obvious but it represents a fundamentally different entry point to other urban food discourses which seek to create a distinct food structure at the urban scale (see Haysom, 2015).

Planning and urban design are not necessarily the same thing where planning has a greater policy alignment to that of urban design. This tension is evident in emerging debates on Water Sensitive Urban Design or Water Sensitive Planning. Water Sensitive Design as this enables wider watershed considerations extending the approaches beyond the limitations of the urban scale. Given this paper’s considerations of the utility of FSPUD in the South African and African contexts, the paper draws on FSPUD as a concept. Due recognition is given to the fact that this merging of disciplines, and scales of governance, could create contextual challenges. It is appreciated that “spatial planning happens at a variety of scales through plans and policies prepared by planners and policy actors, whereas urban design happens at a much smaller, precinct scale but typically, doesn’t directly include policy” (Faragher, 2021). However, both planners and urban designers have not adequately addressed urban food system issues in their practice.

Required is a general transition to Urban Food Planning. Ilieva (2016) suggests that there are four essential practices evident in food system planning. These include conceptual practice, analytical practice, organisational practice and the design practice. This is important for the reason that when used as an organising and urban governance tool, integrating food governance and planning can be seen as being disproportionately aligned to the design component (both urban and policy design), with other essential components lost. Understanding the full extent of the urban food system challenge, assuming responsibility for these issues at the urban scale in the first place, and carefully considering the organisational and governance processes needed to support, and manage, sustained and equitable food system change is key to the FSPUD concept. These different practices are expanded on in Table 2.

**Table 2:** Urban food planning as a field of conceptual, analytical, design, and organisational practices (source: adapted from Ilieva, 2016: 16)

Urban food system planning and design practices	Key practice related questions
Conceptual	Why should we care? What is our obligation (e.g.: Right to Food)?
Analytical	What is the problem? What is the specific urban problem?
Organisational	Who is in charge? Who has powers? How are powers assigned?
Design	How do we solve it?

FSPUD provides a tool to apply a systemic response to the urbanisation of food security and food system challenges, and the governance thereof, connecting food to urban management and functions. This is important because once plans are implemented; management of the plans requires a different organisational response. In general, South African cities have adequately robust plans but gaps exist in the implementation thereof. There are a number of processes and mandates (and responsibilities) of local government into which food can be inserted. Food Sensitive Planning and Urban Design facilitates the inclusion of other components, in the South African case, informed by the likes of SPLUMA and other land use governance dictates. For FSPUD to hold any viability in South African and African cities, it needs to serve both the formal and informal elements of the food system and food system functioning. As Fig. 1 demonstrated, for many of the poorer households in the Cape Town case, the informal food retail sector is an essential point of food access. Represented in an admittedly linear manner, and accepting that the formal and informal binary is seldom clear cut, Table 3 offers a sense of the extreme differences between formal and informal planning. It also demonstrates how formalised planning may allow for some certainty and systemic engagement, but given the significant role that informal “planning” plays in the food system, this demands very different forms of engagement and planning.

**Table 3:**
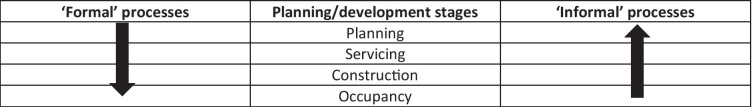
Formal versus informal planning trajectories and processes (source: Battersby, 2019)

In the South African case, the links between the municipal planning competence and food systems are evident but seldom directly considered. In the Cape Town case, the tensions between development and the retention of peri urban farmland (Battersby et al., 2014), the inability of the city to offer details on supermarket expansion and the locating of informal vending areas in well-located sites (Peyton et al., 2015) all demonstrate sites where the municipal planning competence and food systems intersect. These examples also highlight how an absent food planning view can have unintended consequences (Pothukuchi & Kaufman, 1999). Examples of sites where FSPUD can be applied, but also, where the intersections between formal and informal planning processes intersect are evident in sites such as taxi ranks and transit interchanges, where formal planning intersect with informal retail (Park Ross, 2019), as well as in collaboratively organising and structuring places of food trade (Dobson & Skinner, 2009).

This presents a real operational challenge to planners and other urban governance actors. Context, politics, urban needs and the everyday actions of planners and urban managers need to engage the questions, even tensions, of insurgent (informal) planning and structured (formal) planning in ways that maximise benefits and governance for all. This is a clearly understood Southern urban challenge and one through which food offers some opportunities.

##  Conclusion

It would be incorrect to say that Food Sensitive Planning and Urban Design is a trend. FSPUD is still in its infancy, emerging as a new way in which to de-scale and integrate food into local government planning and design. Despite being a persistent issue, the drastic lessons being learnt through the SARS-CoV-2 pandemic, demand new ways of urban provisioning and governance. Urban food governance and planning needs have been largely ignored in urban planning but are now seen to be seen as central to any aspirations of “building back better”.

Perhaps the overriding challenge faced by South African cities, specifically the food insecure and hungry residents of these cities, is the fact that South African city managers believe that they have no mandate to govern food system–related activities. This is a false perception. The Constitution and other planning directives following from the Constitution make a proactive (or progressive) engagement in such food system activities an obligation of local and provincial governments. Initiating “silver bullet” projects such as urban agriculture initiatives can no longer be deemed appropriate. In fact, unless such projects form part of a detailed strategic urban scale food system plan and planning process, they serve as nothing more than politicking and a dereliction of city official’s and politician’s obligation to society.

Importantly, cities, particularly the larger metropolitan areas, are currently engaging multiple contemporary challenges, restructuring operating systems and processes to engage these challenges. Many of these challenges also demand greater integration and coordination. By their very nature, they require that city officials and politicians find ways to connect disparate initiatives and governance fragmentation. Issues such as climate change, resilience, migration, and global reputation all demand engagement across sectors, governmental silos and skills. Food systems planning is similar and forms part of a new type of urban governance.

## Data Availability

The Hungry Cities Partnership data is available at the DataFirst Repository at https://www.datafirst.uct.ac.za/dataportal/index.php/catalog/HCP (released 01 March 2021).

## Footnotes

1 The 2019 Food Poverty Line is R561 (using April 2019 prices) per person per month, from R547 in the 2018 year. That is about R18.70 a day.

2 A “choke” is a controversial water management device (WMD) programmed to provide a daily allocation of water for use per property. The device switches on at a fixed time each morning (e.g. 4 am) and generally restricts water to 350 l a day and 10.5 kl a month—although a different amount can be agreed between you and the City. It switches off when the household has used its daily water quota. It resets the next morning to release the daily amount. See: https://www.capetown.gov.za/Family%20and%20home/residential-utility-services/residential-water-and-sanitation-services/water-management-devices.
